# Differences in the Gastric Microbiotas of Neighboring Populations With Divergent Risks of Gastric Cancer

**DOI:** 10.1111/jgh.70587

**Published:** 2026-07-21

**Authors:** Juan Camilo Caguazango, Karla Vinasco, Isidora Simovic, Yomaira Yépez Caicedo, Marino Coral Bedoya, Nadeem O. Kaakoush, Natalia Castaño‐Rodríguez

**Affiliations:** ^1^ School of Biotechnology and Biomolecular Sciences University of New South Wales Sydney Australia; ^2^ Centro de Enfermedades Digestivas y Nutricionales CIEDYN Pasto Colombia; ^3^ Programa de Nutricion y Dietetica Universidad Mariana Pasto Colombia; ^4^ School of Biomedical Sciences University of New South Wales Sydney Australia

**Keywords:** gastric dysbiosis, gastric premalignant lesions, *Helicobacter pylori*, lactate, oral pathobiont

## Abstract

**Background:**

Although *Helicobacter pylori* plays a central role in gastric cancer (GC), other microorganisms involved in gastric dysbiosis may also impact carcinogenesis. We aimed to characterize gastric dysbiosis and its role in the transition from non‐atrophic gastritis (NAG) to gastric premalignant lesions (GPLs) in two populations from Nariño, Colombia, with distinct GC risks: low‐risk Afro‐Colombians from Barbacoas and high‐risk Amerindian‐Mestizos from Cumbal.

**Methods:**

Gastric biopsies from 200 individuals were analyzed using 16S rRNA gene sequencing. Microbial diversity and composition were assessed based on relevant clinical and sociodemographic characteristics.

**Results:**

*H. pylori* infection was more prevalent in Barbacoas (83.8%) than Cumbal (70.5%), alongside higher rates of obesity (*p <* 0.001), alcohol intake (*p* : 0.07), and smoking (*p* : 0.02), but less severe OLGIM stages (*p* : 0.04). In Barbacoas, alpha diversity remained stable, whereas in Cumbal, richness significantly increased in GPL (*p* < 0.001). Beta diversity showed significant microbial composition differences between municipalities (*p* < 0.001). In Barbacoas, taxa such as *Prevotella*, *Haemophilus*, and *Veillonella* were dominant, whereas lactic acid bacteria such as *Streptococcus* were predominant in Cumbal. Barbacoas showed stable gastric microbiota composition across histological stages, whereas Cumbal exhibited enrichment of *Gemella*, *Leptotrichia*, and *Prevotella* in GPL.

**Conclusion:**

The *Streptococcus*‐driven dysbiosis in Cumbal may help identify microbial biomarkers for GC progression, particularly other oral pathobionts.

## Graphical abstract

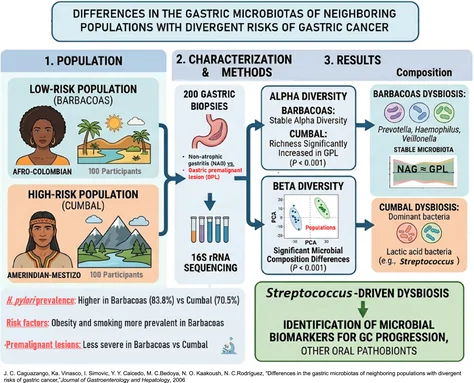

## 1 Introduction

*Helicobacter pylori* , the main risk factor for gastric cancer (GC), coexists with a community of microorganisms that contribute to gastric mucosal homeostasis [1, 2]. Understanding the role and dynamics of this microbiome in intestinal‐type GC continues to rely on Correa's cascade [3, 4], the globally accepted histological framework describing progression from non‐atrophic gastritis (NAG) to atrophic gastritis (AG), intestinal metaplasia (IM), dysplasia (DYS), and ultimately GC [4]. Ecological principles have increasingly been integrated into this continuum, recognizing that disruptions in host–microbiota equilibrium, such as *H. pylori* dominance and reduced gastric acidity, can modify the gastric niche, favoring colonization by opportunistic or pathogenic bacteria and potentially promoting carcinogenesis [5].

The department of Nariño, Colombia, exemplifies the “Colombian enigma,” characterized by marked heterogeneity in intestinal‐type GC incidence despite uniformly high *H. pylori* prevalence [6, 7]. To further elucidate these geographical disparities, we investigated the gastric microbiota and key clinical and sociodemographic features in two contrasting populations: Cumbal, an Amerindian–Mestizo high‐risk community located at 3050 m a.s.l. with an average GC mortality rate of 12.25 per 100 000 inhabitants, and Barbacoas, an Afro‐descendant low‐risk population situated at 36 m a.s.l. in the historically isolated Pacific region, reporting 2.62 GC deaths per 100 000 inhabitants [8].

## 2 Materials and Methods

### 2.1 Field Phase and Recruitment

A total of 200 adults from Cumbal and Barbacoas (Nariño, Colombia) were recruited between November 2021 and April 2022 as part of the URKUNINA 5000 study [9], a community‐based population study designed to investigate the prevalence of gastric premalignant lesions (GPLs) in different municipalities. Participants were recruited from the general population through community outreach and engagement activities rather than through referral for gastrointestinal symptoms or routine clinical care. Sociodemographic and clinical data were obtained through structured interviews, and ethnicity was self‐reported. Gastric biopsies, serum, and plasma were collected during esophagogastroduodenoscopy (EGD), stored at −80°C at the University of Nariño, and later transported to UNSW Sydney.

Adults aged 30–70 years who had resided in the study municipalities for ≥ 10 years were recruited. Exclusion criteria included prior GC or other cancers, recent EGD (< 1 year), and use of antibiotics, bismuth, proton pump inhibitors, NSAIDs, or anticoagulants within the previous month. Individuals with a history of gastric surgery or conditions complicating EGD were also excluded, as detailed in the URKUNINA 5000 protocol [9].

### 2.2 Histological Diagnosis

Five gastric mucosa biopsies (two from the antrum, one from the incisura angularis, and two from the body) were required for histopathological analysis according to the updated Sydney classification system [10]. The diagnostic categories included NAG, AG, IM, and DYS. A modified Giemsa staining was used for the detection of *H. pylori* . Assessment of the gastric biopsies was carried out by the Pathology Department of Hospital San José de Bogotá, Fundación Universitaria de Ciencias de la Salud (FUCS).

### 2.3 Processing and Quality Control of Sequencing Data

Methods for 16S rRNA gene amplicon sequencing are described in Supporting Information. The raw sequencing data were processed using the MiSeq standard analysis pipeline with minor modifications (Mothur v1.48.0 and vsearch v2.22.1) [11]. Further quality control was performed to remove operational taxonomic unit (OTU) contaminants originating from the extraction kit buffers. This was conducted using MicrobIEM (Version 0.7) [12].

### 2.4 Statistical Analysis

Following the processes of exclusion and normalization, a total of 187 participants were included for further analysis. Initially, we evaluated whether the groups exhibited significant differences concerning potential confounding variables. To achieve this, we conducted the chi‐square, Fisher's exact, and Mann–Whitney U tests for all categorical and discrete variables.

### 2.5 Microbial Community Analysis

Alpha diversity metrics, including species richness, Pielou's evenness, and Shannon's index, were computed using Primer‐E v7 software. Group differences based on population and histological status (NAG and GPLs, which included AG, IM, and DYS), as well as *H. pylori* status, were assessed. Data were initially tested for normality using the Shapiro–Wilk test. Normally distributed data were analyzed using an unpaired *t*‐test where group variance did not differ and Welch's *t*‐test where it did. Data that was not normally distributed were analyzed using the Mann–Whitney U test. The data of the gastric microbiota were represented in histograms at the taxonomic level of OTU according to the municipality and therefore, GC risk level. All analyses were performed using GraphPad Prism v10, with a statistical significance threshold set at *p* < 0.05.

For beta diversity analyses, the OTU count table was transformed using the centered log‐ratio (CLR) method with the clr_lite function from the Tjazi package in RStudio Version 4.4.1 [13]. Principal coordinate analysis (PCoA), permutational multivariate analysis of variance (PERMANOVA), and permutational analysis of multivariate dispersions (PERMDISP) were conducted to assess whether significant separation among samples existed by evaluating the effect of factors such as municipality, sex, age, *H. pylori* status, histological diagnosis, ethnicity, alcohol consumption, and smoking. All the previous analyses were based on the Aitchison distances (Euclidean distance between samples after CLR transformation) and calculated with 9999 permutations using the Primer‐E v7 software package.

All beta diversity analyses were validated by calculating Bray–Curtis dissimilarities based on the square root‐transformed relative abundances of OTUs (%). Only variables that showed statistically significant differences according to PERMANOVA were represented in the PCoA plots.

To identify the associations between individual OTUs and variables of interest, we utilized the microbiome multivariable association with linear models (MaAsLin2) through the Galaxy web application provided by the Huttenhower laboratory [14]. This analysis focused on the comparisons between municipalities and considered histological diagnosis and *H. pylori* consensus status as fixed effects. Covariates such as sex, age, ethnicity, BMI, alcohol consumption, and smoking status were included as random effects. The minimum relative abundance for each OTU was set to 0, requiring features to be detected in at least 10% of the samples for inclusion. OTUs were retained based on a variance greater than 0. OTU counts were centered log‐transformed (CLR), with significance assessed using a threshold of *q* < 0.10. Bar plots were generated to visualize the top significant features associated with the fixed effects, complementing the comparison of microbiota profiles between and within the populations.

### 2.6 *H. pylori* Consensus Status

*H. pylori* infection consensus status was determined by integrating histopathology, serology, and 16S rRNA gene sequencing data. Further methods can be found in Supporting Information.

## 3 Results

### 3.1 Characteristics of the Cohorts

Age and sex were comparable across the two studied populations, whereas severe gastric precancerous lesions (i.e., OLGIM III and IV) were more prevalent in the high‐risk population of Cumbal compared with the low‐risk population of Barbacoas.

*H. pylori* infection prevalence was significantly higher in Barbacoas (83.8%) compared with Cumbal (70.5%; *p* = 0.029). Among *H. pylori*‐positive individuals, the proportions of seropositivity for the virulence factors cagA and vacA were similar between high‐ and low‐risk municipalities. CagA positivity was 97.7% in the high‐risk municipality and 98.9% in the low‐risk municipality (*p* = 0.60). Further, the frequency of vacA positivity did not differ significantly between the two groups (88.6% vs. 93.3%; *χ* ^2^ = 1.15; df = 1; *p* = 0.28).

Most participants in Barbacoas were Afro‐Colombians (92.9%), whereas in Cumbal, the population comprised 69.3% Amerindians and 30.7% Mestizo individuals (*p* < 0.05). Comparison of additional clinical and lifestyle characteristics showed statistically significant differences between the two populations, including BMI (*p* < 0.001) and smoking (*p* = 0.02) (see Tables 1 and S1).

**TABLE 1:** Sociodemographic characteristics of the participants from two Colombian populations with opposite risk of GC.

Variable	Barbacoas (LR)	Cumbal (HR)	*p*
	*n* = 99	*n* = 88	
Sex			0.14
Female	73.8%	63.6%	
Male	26.2%	36.4%	
Age^a^ (years)	49.2 ± 9.7	47.3 ± 10.6	0.17
Dx_bivariate			0.47
NAG	68.7%	64.8%	
GPL	31.3%	35.2%	
Dx_Correa's cascade			0.34
NAG	68.7%	64.8%	
AG	2.0%	0.0%	
IM	29.3%	34.1%	
DYS	0.0%	1.1%	
OLGIM	*n* = 29	*n* = 30	**0.04**
I	62.1%	33.3%	
II	20.7%	16.7%	
III	13.8%	26.7%	
IV	3.4%	23.3%	
*H. pylori* consensus status			**0.03**
Negative	16.2%	29.5%	
Positive	83.8%	70.5%	
CagA positivity	98.9%	97.8%	0.60
VacA positivity	93.3%	88.6%	0.28
Ethnicity			**< 0.001**
Amerindian	0.0%	69.3%	
Mestizo	7.1%	30.7%	
Afro‐Colombian	92.9%	0.0%	
BMI			**0.001**
Normal	24.2%	27.3%	
Overweight	26.3%	48.9%	
Obesity	49.5%	23.9%	
Alcohol intake			0.07
No	92.9%	98.9%	
Yes	7.1%	1.1%	
Smoking			**0.02**
No	90.9%	98.9%	
Yes	9.1%	1.1%	

1 Abbreviations: AG: atrophic gastritis, BMI: body mass index, DYS: dysplasia, GPL: gastric premalignant lesion, HR: high risk, LR: low risk, NAG: non‐atrophic gastritis, OLGIM: operative link on gastritis assessment with intestinal metaplasia.

^a^ Mean age ± standard deviation of participants.

### 3.2 Quality Control of Sequencing Data and Assessment of *H. pylori* Dominance

Thirteen samples were excluded from the analysis due to failure to meet quality control criteria. Therefore, the final count table included 187 samples, with an average of 24 545 reads per sample (±14 713 SD). In total, we identified 1465 OTUs from the initial dataset, and 492 OTUs remained after the contaminant removal process.

**FIGURE 1:**
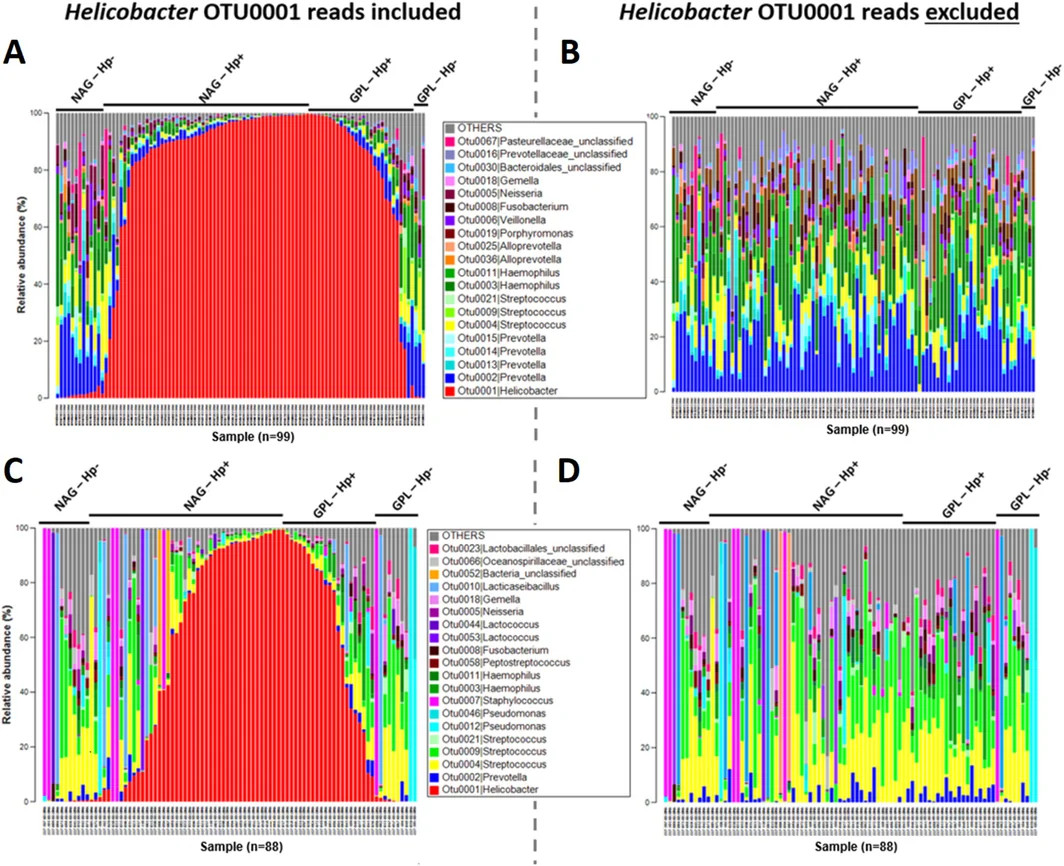
Relative abundances of OTUs according to histological diagnosis and *H. pylori* infection status. (a) Barbacoas (LR), including the contribution of *Helicobacter* OTU0001. (b) Barbacoas (LR), excluding the contribution of *Helicobacter* OTU0001. (c) Cumbal (HR), including the contribution of *Helicobacter* OTU0001. (d) Cumbal (HR), excluding the contribution of *Helicobacter* OTU0001. **GPL–Hp−:** gastric premalignant lesion negative for *H. pylori* . **GPL–Hp+:** gastric premalignant lesion positive for *H. pylori* . **NAG–Hp−:** non‐atrophic gastritis controls negative for *H. pylori* . **NAG–Hp+:** non‐atrophic gastritis controls positive for *H. pylori* .

Given the strong ecological dominance of *H. pylori* [ 15], we examined community profiles with and without *Helicobacter* OTU0001 reads (Figure 1). *H. pylori* showed a higher likelihood of dominance in Barbacoas than in Cumbal (Figure 1a,c). After its removal, the underlying non‐ *H. pylori* microbiota appeared more consistent across populations (Figure 1b,d), highlighting the importance of evaluating diversity both including and excluding this taxon, particularly as *H. pylori* declines along Correa's cascade.

### 3.3 Assessment of the Gastric Microbiota According to GC Risk

**FIGURE 2:**
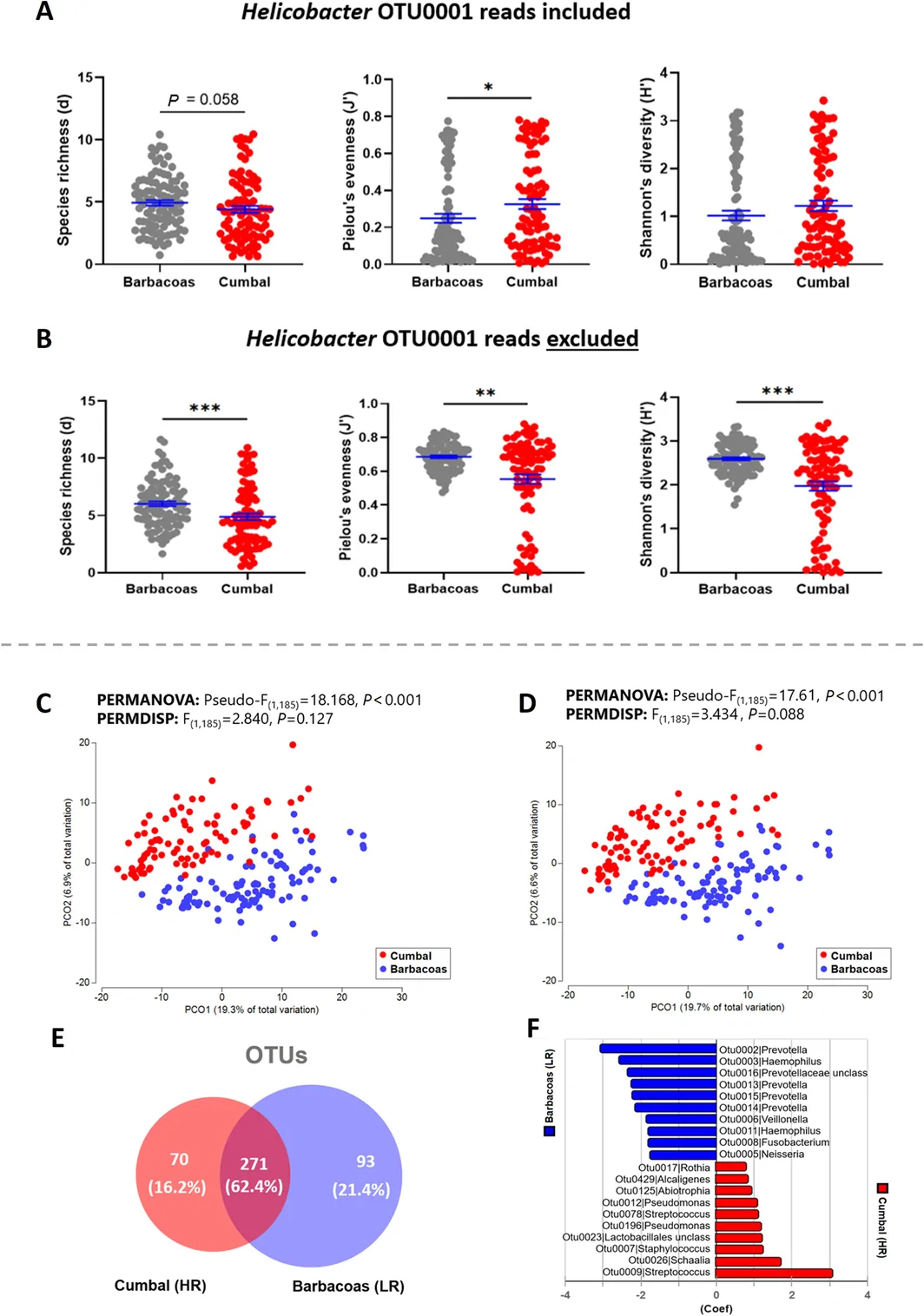
(a, b) Alpha‐diversity metrics between populations with distinct risks of gastric cancer. (a) Diversity metrics comparison including *Helicobacter* OTU0001 reads. (b) Diversity metrics comparison excluding *Helicobacter* OTU0001 reads. The *p*‐values were calculated using the Mann–Whitney U test. * *p* < 0.05, ** *p* < 0.01, ***p < 0.001. (c, d) Principal coordinates analysis (PCoA) according to the municipality with and without *Helicobacter* OTU0001 reads, respectively. The ordination plots were generated based on Aitchison distance (Euclidean distance between samples after centered log‐ratio transformation of the bacterial relative abundances). (e) Venn diagram showing the OTUs shared between the two municipalities. (f) Linear model coefficients for microbial taxa linked to either Barbacoas (*n* = 99) or Cumbal (*n* = 88), adjusted for all sociodemographic and clinical variables included. Negative coefficients indicate an association of microbial species with Barbacoas (blue), whereas positive coefficients values show association with Cumbal (red). The top 10 most representative coefficient values for each municipality with a *q*‐value of less than 0.05 are displayed in bar plots. **Coef:** coefficient, **HR:** high risk, **LR:** low risk.

Alpha diversity metrics revealed greater microbial richness in Barbacoas compared with Cumbal (*p* = 0.058), reaching statistical significance after removal of *H. pylori* reads (Mann–Whitney U test, *p* < 0.001) (Figure 2a,b). In contrast, species evenness was higher in Cumbal when *H. pylori* was present (*p* = 0.015). After removing *H. pylori* , both evenness and Shannon diversity were significantly higher in Barbacoas, consistent with the greater dominance of the bacterium in this population (Table 1; Figure 1a,c). These findings were confirmed when only individuals with NAG were analyzed (Figure S1).

Significant compositional (beta diversity) differences were observed by municipality, age, *H. pylori* status, and histological diagnosis (Figure 2c,d). These associations persisted after removing *H. pylori* reads. Dispersion effects were only noted for histological diagnosis and *H. pylori* status (PERMDISP *p* = 0.017 and *p* < 0.001, respectively). No significant differences were found for sex, ethnicity, BMI, alcohol intake, or smoking (Tables S2 and S3). The analysis remained consistent when Bray–Curtis similarities were employed, both with and without the *Helicobacter* OTU0001 reads (Table S4).

Per‐taxon analyses identified 271 OTUs shared between populations, with 70 specific to Cumbal and 93 to Barbacoas (Figure 2e). Sample sizes used in the between‐population analyses are reported in Table S5. MaAsLin2 identified a total of 67 significant associations between OTUs and municipalities (*q* < 0.10; 59 associations at *q* < 0.05 when corrected for sociodemographic and clinical variables). A summary of the principal differentially abundant taxa identified between municipalities is provided in Table S6. In Barbacoas, the main associations included members of the Prevotellaceae family such as OTU0002 (100% similarity to *Prevotella melaninogenica* ), OTU0016 (88.84% similarity to *Alloprevotella rava* ), OTU0015 (100% similarity to *Hoylesella nanceiensis* , homotypic synonym for *Prevotella nanceiensis* ), OTU0014 (88.84% similarity to *Prevotella pavens* ), *Veillonella* OTU0006 (100% similarity to two species including *Veillonella dispar* ), and *Haemophilus* OTU0003 (100% similarity to *Haemophilus parainfluenzae* ). In Cumbal, notable associations included *Streptococcus* OTU0009 (100% similarity to four *Streptococcus* species), *Schaalia* OTU0026 (100% similarity to *Schaalia odontolytica* ), *Lacticaseibacillus* OTU0010 (100% similarity to *Lacticaseibacillus paracasei* ), *Staphylococcus* OTU0007 (100% similarity to *Staphylococcus aureus* ), and Lactobacillales unclassified OTU0023 (99.6% similarity to *Granulicatella adiacens* ) (Figure 2f, Table S7).

### 3.4 Effects of Age, Histological Diagnosis, and *H. pylori* Infection in the Low‐Risk Population

**FIGURE 3:**
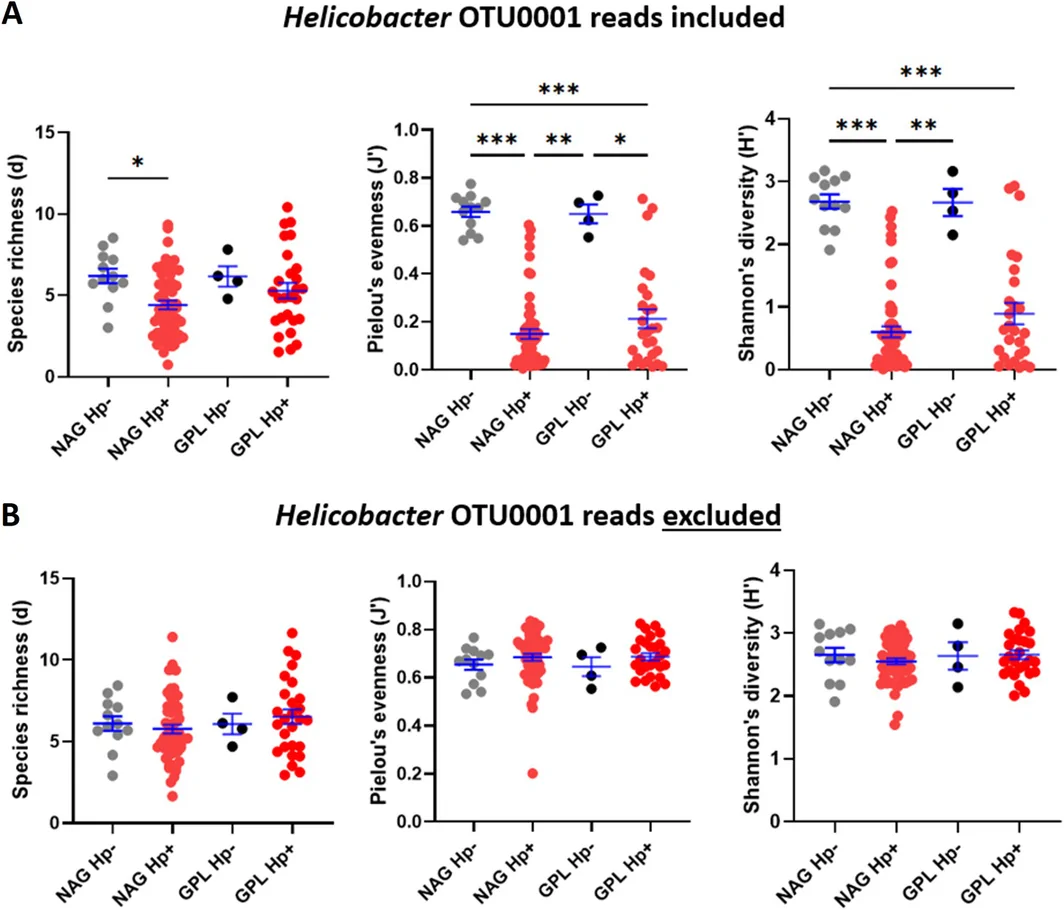
(a) Alpha diversity of the gastric microbiota according to histological diagnosis and *H. pylori* infection status, including *Helicobacter* OTU0001 reads. (b) Alpha diversity of the gastric microbiota according to histological diagnosis and *H. pylori* infection status, excluding *Helicobacter* OTU0001 reads. The *p*‐values for richness were calculated using one‐way ANOVA with a Tukey multiple comparisons test. Other comparisons were made using a Kruskal–Wallis's test with Dunn's multiple comparison test. * *p* < 0.05, **p < 0.01, ***p < 0.001. **GPL–Hp−:** gastric premalignant lesion negative for *H. pylori* . **GPL–Hp+:** gastric premalignant lesion positive for *H. pylori* . **NAG–Hp−:** non‐atrophic gastritis controls negative for *H. pylori* . **NAG–Hp+:** non‐atrophic gastritis controls positive for *H. pylori* .

In Barbacoas, microbial richness was significantly higher in *H. pylori* ‐negative (Hp−) individuals with NAG compared with *H. pylori* ‐positive (Hp+) individuals with NAG (Figure 3a). Both evenness and Shannon diversity were lower in Hp+ individuals, and these differences disappeared after removing *H. pylori* reads (Figure 3b), confirming its strong influence on alpha diversity.

**FIGURE 4:**
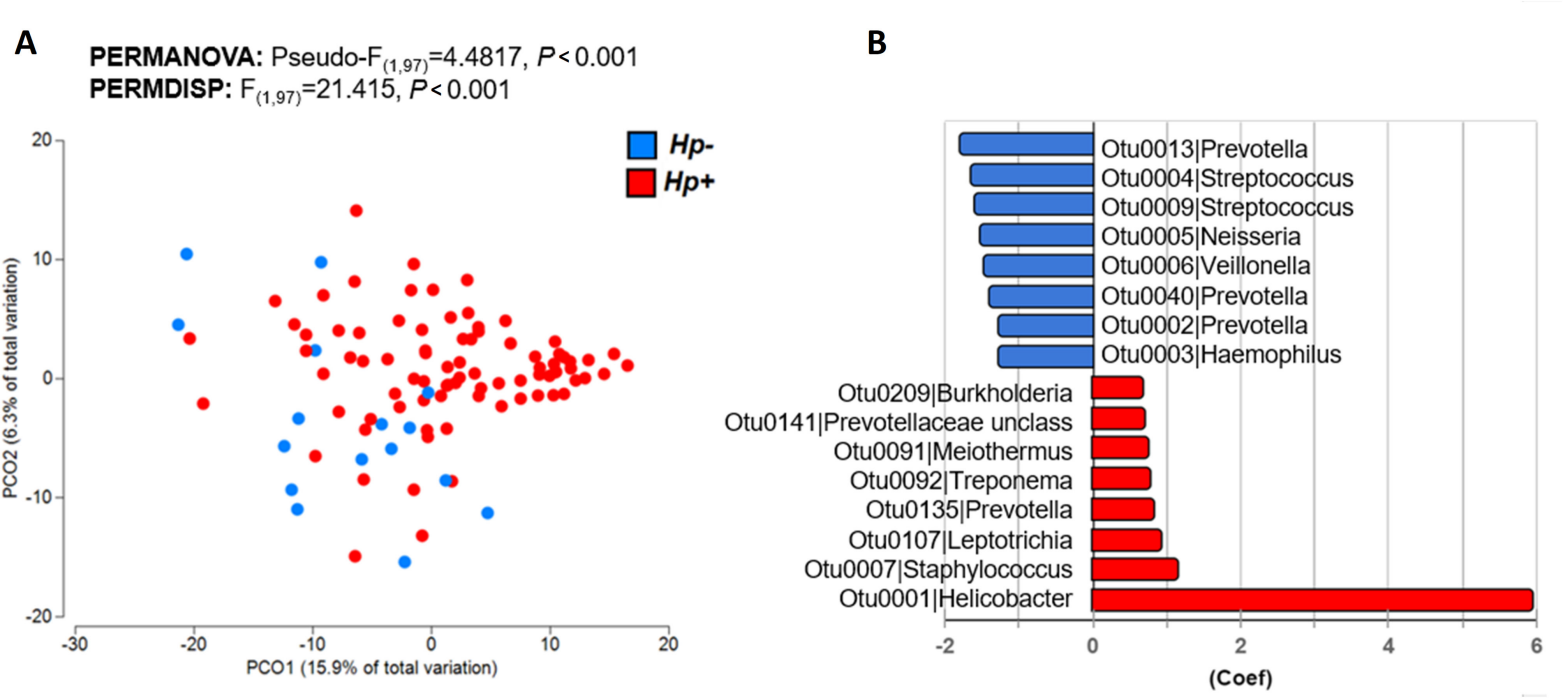
(a) Principal coordinates analysis (PCoA) of bacterial composition in Barbacoas (LR) according to *H. pylori* consensus status. The ordination plot includes *Helicobacter* OTU0001 reads and was generated based on Aitchison distance (Euclidean distance between samples after centered log‐ratio transformation of the bacterial relative abundances). (b) Linear model coefficients for microbial taxa linked to individuals without *H. pylori* infection (*n* = 16) or infected individuals (*n* = 83), adjusted for clinical and sociodemographic factors. Negative coefficients indicate an association of microbial species with the absence of *H. pylori* (blue), whereas positive coefficient values indicate association with the presence of the infection (red). The most representative differential taxa according to *H. pylori* status are displayed in bar plots (*q* < 0.05). MaAsLin2 (microbiome multivariable association with linear models) was used for this analysis. **Hp−:** individuals without *H. pylori* infection, **Hp+:** *H. pylori* infected individuals.

Beta diversity differed by age (*p* < 0.01) and *H. pylori* status (*p* < 0.001), but not by sex, ethnicity, BMI, histological diagnosis, alcohol intake, or smoking (Figure 4a, Table S8). Removal of *H. pylori* reads had minimal impact on these associations (Table S9). In addition, the associations remained consistent in the analysis based on Bray–Curtis similarity, both with and without *Helicobacter* OTU0001 reads (Table S10).

Per‐taxon analyses identified 38 OTU associations with *H. pylori* status (*q* < 0.10; 20 associations at *q* < 0.05), including enrichment of *Staphylococcus* OTU0007, *Leptotrichia* OTU0107 (98.8% similarity to *Leptotrichia wadei* ), and *Treponema* OTU0092 (98.8% similarity to *Treponema denticola* ) in Hp+ individuals, whereas *Prevotella* sp. and *Streptococcus* sp. were more abundant in Hp− samples (Figure 4b, Table S11). Sample sizes used in the within‐population analyses are reported in Table S5. No significant associations were identified with histological diagnosis.

### 3.5 Effects of Age, Histological Diagnosis, and *H. pylori* Infection in the High‐Risk Population

**FIGURE 5:**
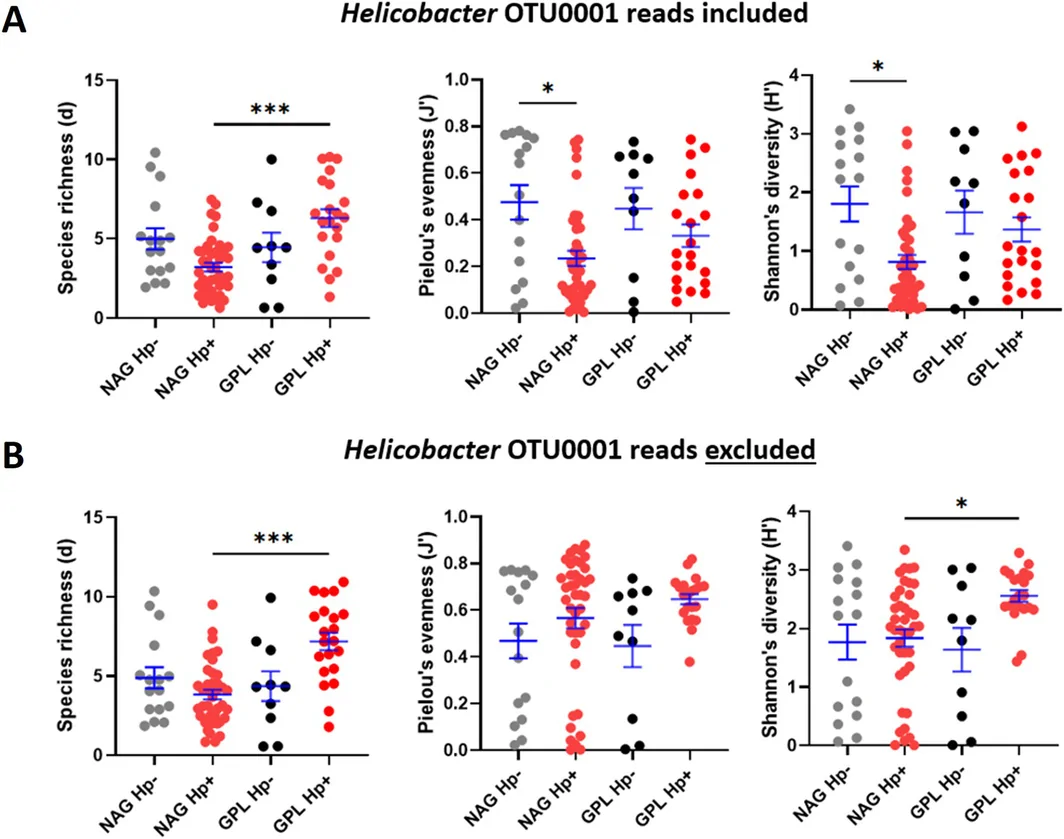
(a) Alpha diversity of the gastric microbiota according to histological diagnosis and *H. pylori* infection status, including *Helicobacter* OTU0001 reads. (b) Alpha diversity of the gastric microbiota according to histological diagnosis and *H. pylori* infection status, excluding *Helicobacter* OTU0001 reads. The *p*‐values for the comparisons were calculated using the Kruskal–Walli's test with a Dunn's multiple comparison test. *p < 0.05, **p < 0.01, ***p < 0.001. **GPL–Hp−:** gastric premalignant lesion negative for *H. pylori* . **GPL–Hp+:** gastric premalignant lesion positive for *H. pylori* . **NAG–Hp−:** non‐atrophic gastritis controls negative for *H. pylori* . **NAG–Hp+:** non‐atrophic gastritis controls positive for *H. pylori* .

In Cumbal, species richness was higher in individuals with GPL Hp+ compared with those with NAG Hp+ (Figure 5a). Conversely, both species evenness and Shannon's index were significantly lower in NAG Hp+ individuals (Figure 5a). When *H. pylori* reads were excluded, the differences in species richness persisted; however, the distinctions in evenness and Shannon diversity between NAG Hp− and Hp+ individuals disappeared. Notably, Shannon diversity remained higher in GPL Hp+ individuals compared with NAG Hp+ individuals (Figure 5b).

**FIGURE 6:**
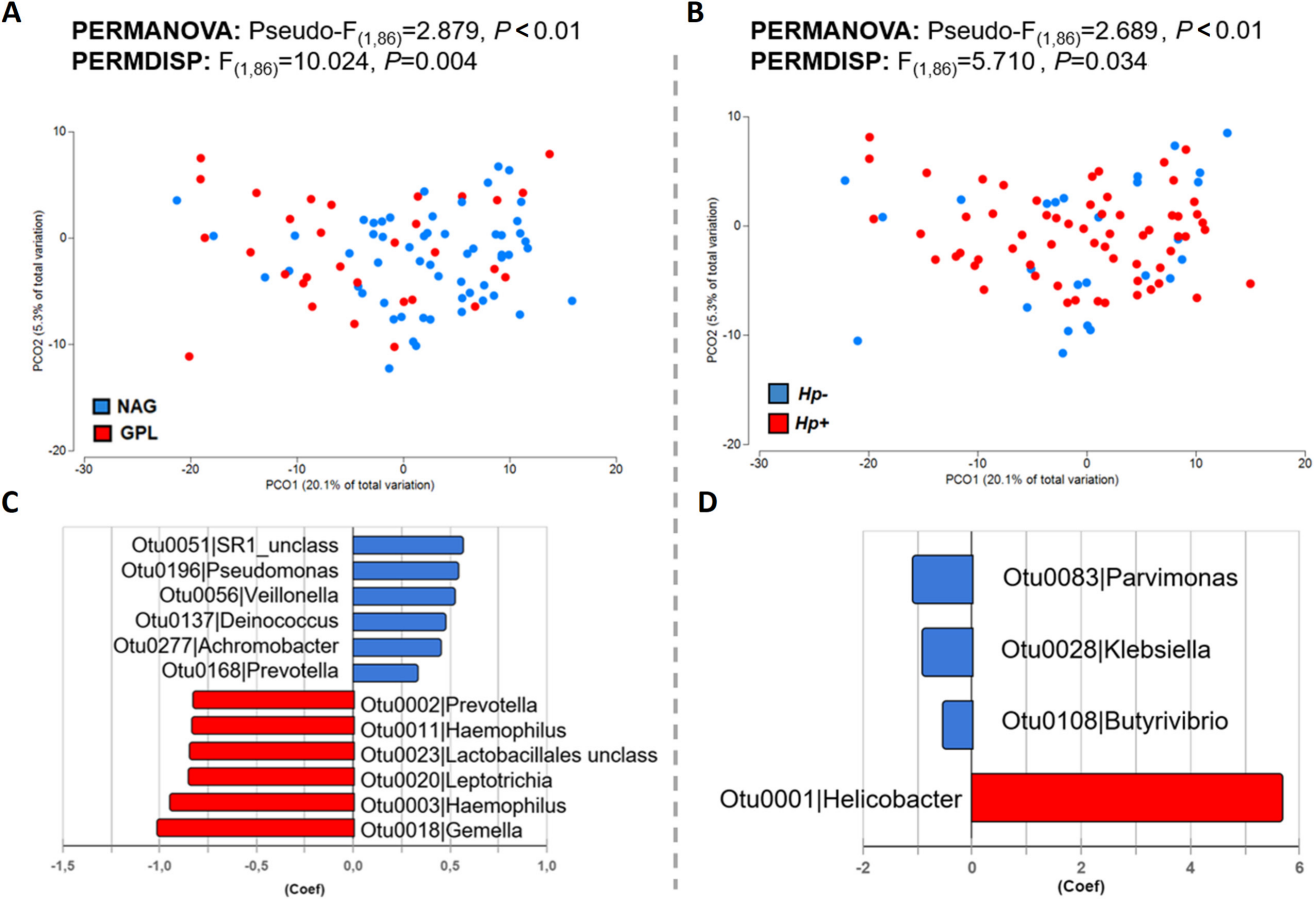
(a, b) Principal coordinates analysis (PCoA) of bacterial composition in Cumbal (HR) according to histological diagnosis and *H. pylori* consensus status. Both ordination plots include *Helicobacter* OTU0001 reads and were generated based on Aitchison distance (Euclidean distance between samples after centered log‐ratio transformation off the bacterial absolute abundances). (c) Linear model coefficients for microbial taxa associated with histological diagnosis of NAG (*n* = 57) or GPL (*n* = 31), accounting for all included variables, are presented in bar plots (*q* < 0.10). Positive coefficients indicate an association with NAG (blue), whereas negative coefficients reflect an association with GPL (red). (d) Linear model coefficients for microbial taxa associated with *H. pylori* consensus status in uninfected individuals (*n* = 26) or infected individuals (*n* = 62), adjusting for all included variables, are shown in bar plots (*q* < 0.10). Negative coefficients indicate an association with the absence of *H. pylori* (blue), whereas positive coefficients indicate an association with *H. pylori* infection (red). The analysis was performed using MaAsLin2. **GPL:** gastric premalignant lesions; **Hp−:** *H. pylori* negative; **Hp+:** *H. pylori* positive; **NAG:** non‐atrophic gastritis.

Beta diversity differed significantly by age, histological diagnosis, and *H. pylori* status (Figure 6a,b). No significant differences in microbial composition were detected for sex, ethnicity, BMI, alcohol intake, or smoking (Table S12). Removal of *Helicobacter* OTU0001 did not alter the factors identified as significant by PERMANOVA (Table S13). Using Bray–Curtis dissimilarity, only age and *H. pylori* status were significant; however, after excluding *H. pylori* reads, these associations were lost, and histological diagnosis became significant instead (Table S14).

MaAsLin2, adjusted for all sociodemographic and clinical variables (Table 1), identified six OTUs significantly associated with NAG (*q* < 0.10; three associations at *q* < 0.05), including *Aliiroseovarius* OTU0128 (100% similarity to *Aliiroseovarius halocynthiae* ), *Endozoicomonadaceae* OTU0109 (96.4% similarity to *Sansalvadorimonas verongulae)*, and *Halodesulfovibrio* OTU0182 (99% similarity to *Halodesulfovibrio oceani* ) (Figure 6c; Table S15). Three OTUs were enriched in GPL (*q* < 0.10; one association at *q* < 0.05), notably *Gemella* OTU0018 (100% similarity to up to five *Gemella* species), *Leptotrichia* OTU0020 (98.8% similarity to *L. wadei* ), and *Prevotella* OTU0031 (100% similarity to *Segatella oris,* homotypic synonym for *Prevotella oris* ) (Figure 6c; Table S15).

In relation to *H. pylori* status, Hp− individuals showed enrichment of *Parvimonas* OTU0083 (100% similarity to *Parvimonas micra* ), *Klebsiella* OTU0028 (100% similarity to *Klebsiella aerogenes* ), and *Butyrivibrio* OTU0108 (96.4% similarity to *Butyrivibrio hungatei* ) (*q* < 0.10; Table S16), whereas *Helicobacter* OTU0001 was the only OTU significantly associated with infection (*q* < 0.001) (Figure 6d; Table S16). Sample sizes used in the within‐population analyses are reported in Table S1.

## 4 Discussion

We examined the gastric microbiota in two populations with distinct risk profiles for GC. Despite both showing a high prevalence o*f H. pylori* infection, notable differences in sociodemographic and clinical factors were observed between the low‐risk Barbacoas population and the high‐risk Cumbal population. Barbacoas residents had higher rates of *H. pylori* infection, smoking, alcohol consumption, and obesity, yet exhibited a lower severity of gastric lesions compared with Cumbal. These findings suggest a complex interplay between microbial ecology, host factors, and environmental conditions influencing gastric disease progression.

A strong inverse association between *H. pylori* abundance and alpha diversity was observed in both municipalities, consistent with previous reports [16, 17]. Furthermore, significant differences in alpha and beta diversity were identified between the populations, irrespective of analytical methods or adjustment for potential confounders. The higher richness and evenness observed in Barbacoas, despite the presence of conventional risk factors, indicate that increased microbial diversity may play a protective role in preventing the transition from gastritis to more advanced lesions.

The greater diversity and stability of the Barbacoas microbiota, coupled with the presence of taxa such as *Prevotella*, *Haemophilus*, and *Veillonella*—commensals typically associated with healthy gastric conditions [15, 17, 18, 19] and generation of short‐chain fatty acids via lactate fermentation [20, 21, 22, 23]—suggest a balanced microbial ecosystem potentially resilient to pathogenic disruption. Microbial composition was not significantly influenced by factors commonly linked to GC risk, such as BMI [24], alcohol intake, or smoking [25], supporting the notion that intrinsic microbial dynamics may contribute to protection against disease progression. Although some of these genera have been associated with gastric disorders [26, 27, 28], their precise pathogenic potential remains unclear.

In contrast, the high‐risk population of Cumbal exhibited lower microbial richness and diversity, along with a notable presence of lactic acid bacteria (LAB) compared with Barbacoas. Enrichment of LAB and accumulation of their by‐products such as microbial lactate, reactive oxygen species, and nitroso compounds have been associated with carcinogenic potential [29]. Although the direct implications of lactate in GC remain uncertain, its ecological impact in a dysbiotic niche could favor colonization by other carcinogenic pathobionts [29].

*Streptococcus* OTU0009 was the most frequent OTU, identified in 87 of 88 individuals, highlighting its central role in the gastric ecosystem. *Streptococcus* species are recognized gastric residents and have repeatedly been reported among the most abundant non‐*Helicobacter* taxa in patients with GC and premalignant gastric lesions [30, 31]. Additional sequence verification demonstrated that OTU0009 matched multiple *Streptococcus* species with identical V4 sequences, precluding reliable species‐level assignment ( *S. ilei* , *S. rubneri* , *S. mobilis* , *S. parasanguinis* ). Interestingly, *S. anginosus* is another *Streptococcus* species that has been recently strongly linked to gastric tumorigenesis in mice through activation of mitogen‐activated protein kinase signalling [32]. *S. anginosus* can alter gastric pH and promote dysbiosis, enriching pathobionts while reducing beneficial taxa, thus creating an inflammatory microenvironment conducive to cancer [32]. Within this framework, *Streptococcus* species could function as “passenger” organisms in the driver–passenger model, similar to *Fusobacterium* in colorectal cancer [33], potentially exacerbating disease progression once a permissive gastric environment has been established.

In Cumbal, microbial diversity was positively associated with gastritis severity, with species richness increasing as lesions progressed. This pattern suggests that microbial diversity may be shaped not only by *H. pylori* but also by other microorganisms and gastric conditions, reflecting a dynamic ecosystem where adaptive microbial mechanisms emerge [5, 34].

We also observed higher abundance of *Gemella* (OTU0018), *L. wadei* (OTU0020), and *P. oris* (OTU0031) in individuals with GPL from Cumbal. These genera, part of the oral microbiota, can access the stomach via saliva and act as commensals with opportunistic potential [34, 35]. In premalignant gastric conditions, *Gemella* and *Leptotrichia* are consistently enriched [35, 36, 37, 38]. Interestingly, *L. wadei* was also prevalent in Tuquerres, another high‐risk municipality in Colombia [7], suggesting a shared microbial signature in high‐risk regions. The recurrence of similar microbial patterns across independent high‐risk populations may indicate that common environmental, dietary, host, and microbial factors contribute to GC risk within this region. *P. oris* , often found in dysbiotic biofilms associated with periodontal disease, has been detected as enriched in both oral and gastric samples of GC patients [39].

An important consideration when interpreting these findings is the substantial environmental contrast between the two study populations. Cumbal is located at high altitude (> 3000 m above sea level), whereas Barbacoas is situated near sea level. Chronic exposure to hypobaric hypoxia may influence gastric physiology through changes in mucosal oxygenation, epithelial metabolism, acid secretion, and host immune responses [40, 41]. Experimental evidence has also demonstrated that hypoxic exposure can alter gastric microbial diversity and composition, including enrichment of Firmicutes‐associated taxa and LAB [40, 41]. However, physiological markers of hypoxic adaptation were not available in the present study and therefore the contribution of chronic hypobaric hypoxia to the observed microbial differences could not be directly evaluated.

Previous studies have documented marked dietary differences between high‐altitude Andean communities such as Tuquerres and low‐altitude Pacific coastal populations such as Tumaco, municipalities that share epidemiological and geographic characteristics with Cumbal and Barbacoas, respectively. High‐altitude populations generally consume diets richer in starchy tubers, dairy products, and salt‐preserved foods, whereas coastal populations have greater access to fruits, vegetables, and seafood [6, 42]. These dietary differences may contribute to the distinct microbial communities observed in the present study. For example, the greater abundance of *Prevotella* observed in Barbacoas may be consistent with dietary patterns enriched in plant‐derived substrates, whereas the predominance of Firmicutes‐associated taxa in Cumbal may be influenced by differences in dietary and environmental exposures. However, dietary intake was not directly assessed in the present study and therefore these interpretations should be considered exploratory.

*H. pylori* prevalence was higher in Barbacoas (LR), whereas antibodies against CagA and VacA were comparable across both populations. The influence of *H. pylori* virulence factors on the gastric microbiome is only beginning to be understood [40]. Colonization by strains expressing CagA, VacA, and BabA has been shown to reduce alpha diversity and alter microbial composition [43, 44]. Further research should aim to characterize the full *H. pylori* virulence profile in these populations and its impact on microbial community structure.

An alternative explanation for the lower abundance of oral‐associated taxa in Barbacoas is that the greater prevalence of *H. pylori* may exert ecological pressure through niche occupation, resource competition, or host‐mediated mechanisms, potentially limiting colonization by microorganisms such as *Streptococcus*, *Gemella*, and *Leptotrichia*. Although our cross‐sectional design does not allow this hypothesis to be directly tested, future studies should investigate potential ecological interactions between *H. pylori* and other members of the gastric microbiota.

The interpretation of beta‐diversity differences should consider the significant dispersion effects observed for histological diagnosis and *H. pylori* status. Under these circumstances, PERMANOVA significance may partially reflect differences in within‐group variability rather than exclusively differences in group centroids. Furthermore, some subgroup sizes, particularly within the Cumbal GPL cohort, were relatively small after stratification by clinical variables. Therefore, the results of within‐population analyses should be interpreted cautiously and warrant validation in larger independent cohorts.

In conclusion, our findings suggest that the low‐risk profile of Barbacoas may result from a diverse, balanced microbiota dominated by “lactate‐eating” taxa that provide resilience against GC progression. Conversely, in Cumbal, dysbiosis characterized by LAB enrichment and other potential pathobionts such as *Leptotrichia, Gemella,* and *Prevotella* may create a microenvironment conducive to carcinogenesis. These microbial patterns should be interpreted within the broader context of environmental, dietary, and host‐related factors that differ substantially between the study populations. Together, these findings underscore the multifactorial nature of GC, highlighting the need to integrate microbial, environmental, and host factors in prevention strategies.

## Funding

N.C‐R. was supported by the UNSW Scientia Fellowship program, NHMRC Investigator Grant Emerging Leadership 2 (EL2) (APP 2033415), a Tour de Cure Mid‐Career Research Grant (RSP‐525‐FY2023), and a Cancer Australia/Pancare Foundation PdCCRS Early Career Researcher Grant (2012944). N.O.K. was supported by a UNSW Scientia Fellowship. J.C.C. was supported by a scholarship from La Fundación Centro de Estudios Interdisciplinarios Básicos y Aplicados (CeiBA Foundation), BECATE Gobernación de Nariño. K.V. and I.S. were supported by an Australian Government Research Training Program (RTP) Scholarship (DOI: https://doi.org/10.82133/C42F‐K220).

## Ethics Statement

All participants signed an informed consent. The present study was approved by the human ethics committees at the Hospital Universitario Departamental de Nariño in Pasto, Colombia, and the University of New South Wales (UNSW), Sydney, Australia (HC210565).

## Conflicts of Interest

The authors declare no conflicts of interest.

## Supporting information


**Table S1:** Sociodemographic characteristics of the participants according to the population.
**Table S2:** Overall PERMANOVA analysis with *Helicobacter* OTU0001 reads (Aitchison distance).
**Table S3:** Overall PERMANOVA analysis without *Helicobacter* OTU0001 reads (Aitchison distance).
**Table S4:** Overall PERMANOVA analysis with and without *Helicobacter* OTU0001 reads (Bray–Curtis similarity).
**Table S5:** Sample sizes used in between‐ and within‐population in MaAslyn analyses.
**Table S6:** Summary of the principal differentially abundant taxa identified between municipalities by MaAsLin2.
**Table S7:** Maaslin2 analysis by municipality.
**Table S8:** Barbacoas—PERMANOVA analysis with *Helicobacter* OTU0001 reads (Aitchison distance).
**Table S9:** Barbacoas—PERMANOVA analysis without *Helicobacter* OTU0001 reads (Aitchison distance).
**Table S10:** Barbacoas—PERMANOVA analysis with and without *Helicobacter* OTU0001 reads (Bray–Curtis similarity).
**Table S11:** Barbacoas—MaAslin2 analysis by *H. pylori* consensus status.
**Table S12:** Cumbal—PERMANOVA analysis with *Helicobacter* OTU0001 reads (Aitchison distance).
**Table S13:** Cumbal—PERMANOVA analysis without *Helicobacter* OTU0001 reads (Aitchison distance).
**Table S14:** Cumbal—PERMANOVA analysis with and without *Helicobacter* OTU0001 reads (Bray–Curtis similarity).
**Table S15:** Cumbal—MaAslin2 analysis by histological grouping NACG‐GPL.
**Table S16:** Cumbal—MaAslin2 analysis by *H. pylori* consensus status.

**Figure S1:** Alpha‐diversity metrics between individuals with non‐atrophic gastritis from populations with distinct risks of gastric cancer. (a) Diversity metrics comparison including *Helicobacter* OTU0001 reads. (b) Diversity metrics comparison excluding *Helicobacter* OTU0001 reads. The *p*‐values were calculated using the Mann–Whitney U test. ** *p* < 0.01 *** *p* < 0.001. **NAG:** non‐atrophic gastritis, **LR:** low risk, **HR:** high risk.

## Data Availability

16S rRNA gene amplicon sequencing data was generated in this paper and has been deposited in the European Nucleotide Archive (PRJEB84002). This can be accessed using the following link: https://www.ebi.ac.uk/ena/browser/view/PRJEB84002.

## Footnotes

1
*Note:* Bold emphasis in *p*‐values denotes statistical signficance.
