# Supplementary material for: Differences in the Gastric Microbiotas of Neighboring Populations With Divergent Risks of Gastric Cancer
Source: J Gastroenterol Hepatol. 2026 Jul 21;41(9):2739–50. doi: 10.1111/jgh.70587 (PMC13534255; doi:10.1111/jgh.70587)
Supplement: Supplementary file 1 [file JGH-41-2739-s002.docx]

**Differences in the gastric microbiotas of neighboring populations with divergent risks of gastric cancer**

1. Supplementary methods
2. Supplementary figure S1
1. **Supplementary methods**

***16S rRNA gene amplicon sequencing***

Genomic DNA was extracted from gastric antrum biopsies preserved at -80 ºC, following a modified optimized protocol published by Peña-Llopis and Brugarolas^1^. DNA extraction kit buffers were further used as negative controls.

The V4 region of the 16S rRNA gene was amplified by PCR using the primers 515 F (5´- GTGCCAGCMGCCGCGGTAA -3´) and 806 R (5´- GGACTACHVGGGTWTCTAAT -3´)^2^, the kit HotStarTaq Plus Master Mix (Qiagen^®^, EE.UU) and the following conditions: 95 °C for 3 min, followed by 25 cycles of 95 °C for 30 s, 55 °C for 30 s and 72 °C for 30 s, after which a final elongation step at 72 °C for 5 min occurred. Normalized and purified PCR products were used to prepare a DNA library using the MiSeq reagent kit V4 (2x250 bp) for paired-end reads on a MiSeq platform according to the manufacturer’s guidelines, at the Ramaciotti Center for Genomics (UNSW Sydney, Australia).

***Helicobacter pylori* consensus status**

Histopathology, conducted according to the updated Sydney system, is considered the gold standard for directly assessing the presence and load of *H. pylori*. An individual was classified as positive if *H. pylori* was detected in at least three out of five biopsies. In cases where histopathology results were inconclusive, such as detection in fewer biopsies or a low bacterial load, additional tests were employed. This included serology, measuring immunoglobulin G and M antibodies indicative of past and current infection, using the Helico Blot 2.1 kit (CE - MP Biomedicals)^3^, and 16S rRNA sequencing data with a threshold of 4.4% relative abundance^4^.

**Supplementary methods references**

1. Peña-Llopis S, Brugarolas J. Simultaneous isolation of high-quality DNA, RNA, miRNA and proteins from tissues for genomic applications. Nat Protoc. 2013 Nov;8(11):2240-55. doi: 10.1038/nprot.2013.141. Epub 2013 Oct 17. PMID: 24136348; PMCID: PMC4211643.
2. Caporaso JG, Lauber CL, Walters WA, et al. Global patterns of 16S rRNA diversity at a depth of millions of sequences per sample. Proc Natl Acad Sci U S A. 2011 Mar 15;108 Suppl 1(Suppl 1):4516-22. doi: 10.1073/pnas.1000080107. Epub 2010 Jun 3. PMID: 20534432; PMCID: PMC3063599.
3. Veijola L, Oksanen A, Sipponen P, et al. Evaluation of a commercial immunoblot, Helicoblot 2.1, for diagnosis of Helicobacter pylori infection. Clin Vaccine Immunol. 2008 Nov;15(11):1705-10. doi: 10.1128/CVI.00165-08. Epub 2008 Sep 30. PMID: 18827192; PMCID: PMC2583517.
4. Gantuya B, El Serag HB, Saruuljavkhlan B, et al. Advantage of 16S rRNA amplicon sequencing in Helicobacter pylori diagnosis. Helicobacter. 2021 Jun;26(3):e12790. doi: 10.1111/hel.12790. Epub 2021 Feb 17. PMID: 33596345; PMCID: PMC8122063.
2. **Supplementary Figure S1**

**Figure S1.** Alpha-diversity metrics between individuals with non-atrophic gastritis from populations with distinct risks of gastric cancer. **(a)** Diversity metrics comparison including *Helicobacter* OTU0001 reads. **(b)** Diversity metrics comparison excluding *Helicobacter* OTU0001 reads. The *P*-values were calculated using the Mann–Whitney U test. *******P* < 0.01 ********P* < 0.001. **NAG:** Non-atrophic gastritis, **LR:** Low risk, **HR:** high Risk.
